# Management of Retinitis Pigmentosa Via Wharton’s Jelly-Derived Mesenchymal Stem Cells or Combination With Magnovision: 3-Year Prospective Results

**DOI:** 10.1093/stcltm/szad051

**Published:** 2023-09-15

**Authors:** Emin Ozmert, Umut Arslan

**Affiliations:** Ankara University Faculty of Medicine, Department of Ophthalmology, Ankara, Türkiye; Ankara University Technopolis, Bioretina Eye Clinic, AnkaraTürkiye; Ankara University Technopolis, Bioretina Eye Clinic, AnkaraTürkiye

**Keywords:** retinitis pigmentosa, stemcell, Wharton jelly, umbilical cord, mesenchymal stemcell, electromagnetic stimulation, iontophoresis, Magnovision

## Abstract

To investigate whether the natural progression rate of retinitis pigmentosa (RP) can be decreased with subtenon Wharton’s jelly-derived mesenchymal stem cell (WJ-MSC) application alone or combination with Magnovision.

The study included prospective analysis of 130 eyes of 80 retinitis pigmentosa patients with a 36-month follow-up duration. Patients constitute 4 groups with similar demographic characteristics. The subtenon WJ-MSC-only group consisted of 34 eyes of 32 RP patients as Group 1; the Magnovision-only group consisted of 32 eyes of 16 RP patients as Group 2; the combined management group consisted of 32 eyes of 16 RP patients who received combined WJ-MSC and Magnovision as Group 3; the natural course (control) group consisted of 32 eyes of 16 RP patients who did not receive any treatment were classified as Group 4. Fundus autofluorescence surface area (FAF-field), horizontal and vertical ellipsoid zone width (EZW), fundus perimetry deviation index (FPDI), full-field electroretinography magnitude (ERG-m), and best corrected visual acuity (BCVA) changes were compared within and between groups after 36 months follow up period.

FAF-field delta changes were detected 0.39 mm^2^ in Group 1, 1.50 mm^2^ in Group 2, 0.07 mm^2^ in Group 3 and 12.04 mm^2^ in Group 4 (Δp 4 > 2 > 1 > 3). Horizontal EZW, Vertical EZW, BCVA, and FPDI delta changes were detected Δp 4 > 1,2 > 3. ERG-m delta changes were detected Δp 3 > 1,2,4.

Retinitis pigmentosa characterized by progressive loss of photoreceptors eventually leading to total blindness. The combination of WJ-MSC and Magnovision can significantly slow the progression of the disease in comparison to natural progression rate for 3 years in appropriate cases.

**Trial Registration**: ClinicalTrials.gov, NCT05800301.

## Graphical Abstract

**Figure:**
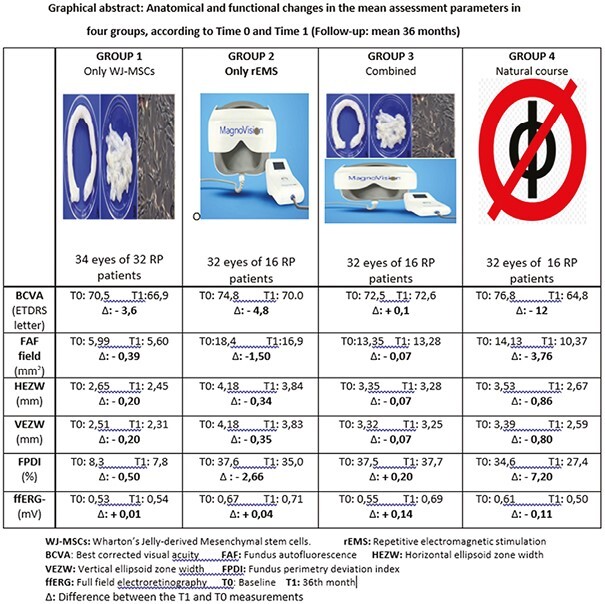

**Significance Statement**

Hereditary retinal dystrophies result in progressive vision loss and total blindness in productive age. In addition to visual impairment, the most important complication is suicide. Injection of Wharton's jelly-derived mesenchymal stem cells into the deep subtenon space is effective and safe for slowing disease progression. Magnovision application, which is used to regularly stimulate exosome degranulation of stem cells and increase the duration of action, has been found to be synergistically effective and safe. A new method that can prevent the progression of retinitis pigmentosa to blindness has been scientifically defined in a 3-year prospective study.

**Lessons Learned**

Retinitis pigmentosa characterized by progressive loss of photoreceptors eventually leading to total blindness. The combination of WJ-MSC and Magnovision can significantly slow the progression of the disease in comparison to natural progression rate for 3 years in appropriate cases.

## Introduction

Retinitis pigmentosa (RP) is one of the most common inherited diseases of retinopathies. It is estimated to affect 1 in 3000 to 1 in 4000 people globally. RP is a genetic disease group characterized by progressive loss of photoreceptors. At least 90 different structural and functional proteins have been identified in the sensory retina, which is necessary for the healthy functioning of the visual cycle. At least 300 genes encode these proteins, and their fragments have been identified in the sensory retina. Mutations in any of these 300 genes lead to outer retinal degeneration and RP. In classical RP, genetic mutations primarily impair the functions of rod cells. Structural and functional protein deficiency causes rod cells to enter the dormant phase and undergo apoptosis. The inheritance pattern can be autosomal dominant, autosomal recessive, X-linked, mitochondrial, or spontaneous mutations. The rate of disease progression is different in each inheritance pattern. Patients first complain of difficulty seeing at night and prolonged dark adaptation. As rod cell loss increases, the peripheral visual field begins to narrow. The narrowing of the visual field progresses at a rate of 5%-15% each year, depending on the inheritance pattern, and finally, the cone cells are affected. Apoptosis of rod/cone cells results in end-stage RP, then progresses to total blindness.^1-3^

Wharton’s jelly-derived mesenchymal stem cells (WJ-MSCs) have a high paracrine effect and secrete exosomes containing different growth factors (GFs) and neurotrophins. These peptides in the exosome content are functional and structural peptides for neurons. Peptides that cannot be encoded in RP can be substituted by WJ-MSCs exosomes. Growth factors and neurotrophins in the exosome can accelerate the entry of glucose into retina pigment epithelium (RPE) and photoreceptors and their conversion to ATP, an energy molecule. These neurotrophins can also provide homeostasis, preventing apoptosis by accelerating the phagocytosis of cellular metabolic wastes.^4-8^

High-frequency repetitive electromagnetic stimulation (rEMS) can modulate ion channels in neurons depending on frequency, magnetic field, and duration variables. If the dormant phase—which is the sleep mode caused by genetic mutations in the sensory retina—is prolonged, apoptosis and permanent photoreceptor loss occur. Activation of ion channels and acceleration of neuromodulation by electromagnetic stimulation can prevent neuronal apoptosis. Scientific studies have also shown that rEMS increases mesenchymal stem cells’ exosome degranulation. Another effect of rEMS is the iontophoresis effect. The passage of large molecules into the cells through the scleral pores is possible by changing the electrical charges between neurotrophins and their receptors and increasing the affinity. It can also induce the delivery of higher amounts of GFs and neurotrophins into the subretinal environment and retina.^9-16^

This prospective clinical study aims to investigate whether RP progression can be slowed or maintained with the inoculation of WJ-MSCs alone into the deep subtenon space or in conjunction with rEMS application compared to the natural course of the disease.

## Materials and Methods

Ethics committee approval for the umbilical cord WJ-MSCs study was obtained from the Ankara University Faculty of Medicine Clinical Research Ethics Committee (17-700-19). It was also approved by the “Review Board of the Cell, Organ, and Tissue Transplantation Department” within the Turkish Ministry of Health (56733164/203 E.2140). Ethics committee approval for the transcranial electromagnetic stimulation study was obtained from the Ankara University Faculty of Medicine Clinical Research Ethics Committee (11-962-19) and the “Review Board of the Drug and Medical Device Department” within the Turkish Ministry of Health (2019-514). The study was performed following the tenets of the 2013 Declaration of Helsinki. Written informed consent was obtained from the RP patients before enrollment.

This prospective, sequential, open-label clinical study was conducted at Ankara University Faculty of Medicine, Department of Ophthalmology, between January 2019 and December 2022. The study included 130 eyes of 80 RP patients with a 36-month follow-up duration. A genetic mutation RP panel test also confirmed the diagnosis in the study cohort and the clinical and imaging findings. All patients enrolled in this study underwent a complete routine ophthalmic examination, including the best-corrected visual acuity (BCVA) measurement with the early treatment of diabetic retinopathy study (ETDRS) chart (Topcon CC 100 XP, Tokyo, Japan). Structural changes of retinal layers were examined and followed using an optical coherence tomography angiography (OCTA) multimodal imaging device from RTVue XR (Avanti, Optovue) and a fundus autofluorescence (FAF) device (Topcon TRC-NW8F plus). Functional changes in the photoreceptors and outer retinal cells were examined and followed using a retina-tracking, computerized perimetry device (Compass, CenterVue) and digital electroretinography (ERG) device (Diopsys Nova ERG-VEP System/RFA), which is an office-based easy-to-use retinal function analyzer of the retina and visual pathway.

The ellipsoid zone width (EZW) showed a healthy inner and outer segment of photoreceptors and was measured horizontally and vertically by the manual segmentation program of the OCTA device. Since many retinal pathologies, including RP, often lead to RPE dysfunction and lipofuscin accumulation, abnormal autofluorescence patterns on FAF imaging can act as markers for retinal disease. The hyperautofluorescent ring constricts over time, acting as a marker of disease progression. The FAF surface area (FAF-field) was calculated automatically via the special program of the FAF device after marking the horizontal and vertical longest axes of the hyperfluorescent field. The fundus perimetry deviation index (FPDI) records were examined in the 24/2 mode by computerized perimetry records. The FPDI offers data explaining how many of the 100 flashing points can be seen correctly by the patient and what percentage of the visual field can be seen. Magnitude changes of multi-luminance flicker-full field electroretinography (ERG-m) were recorded by the Diopsys retinal function analyzer, which refers to the action potentials and phase deviations recorded from global photoreceptors/outer retinal cells stimulated with different light intensities.

### Genetic Analysis

The diagnosis of RP was made clinically after a complete ophthalmological examination. The patients’ clinical and detailed family histories were obtained. In terms of syndromic RP, systemic symptoms, such as hearing loss, polydactyly, and mental retardation, were investigated. After obtaining clinical data, the patient was referred to the medical genetic clinic. Blood samples were taken from patients and, if necessary, from family members; genetic mutations and inheritance patterns were investigated using a DNA RP panel sequencing method consisting of 90 genes.

### Umbilical Cord Wharton’s Jelly-Derived Mesenchymal Stem Cells Preparation

**Figure 1.**
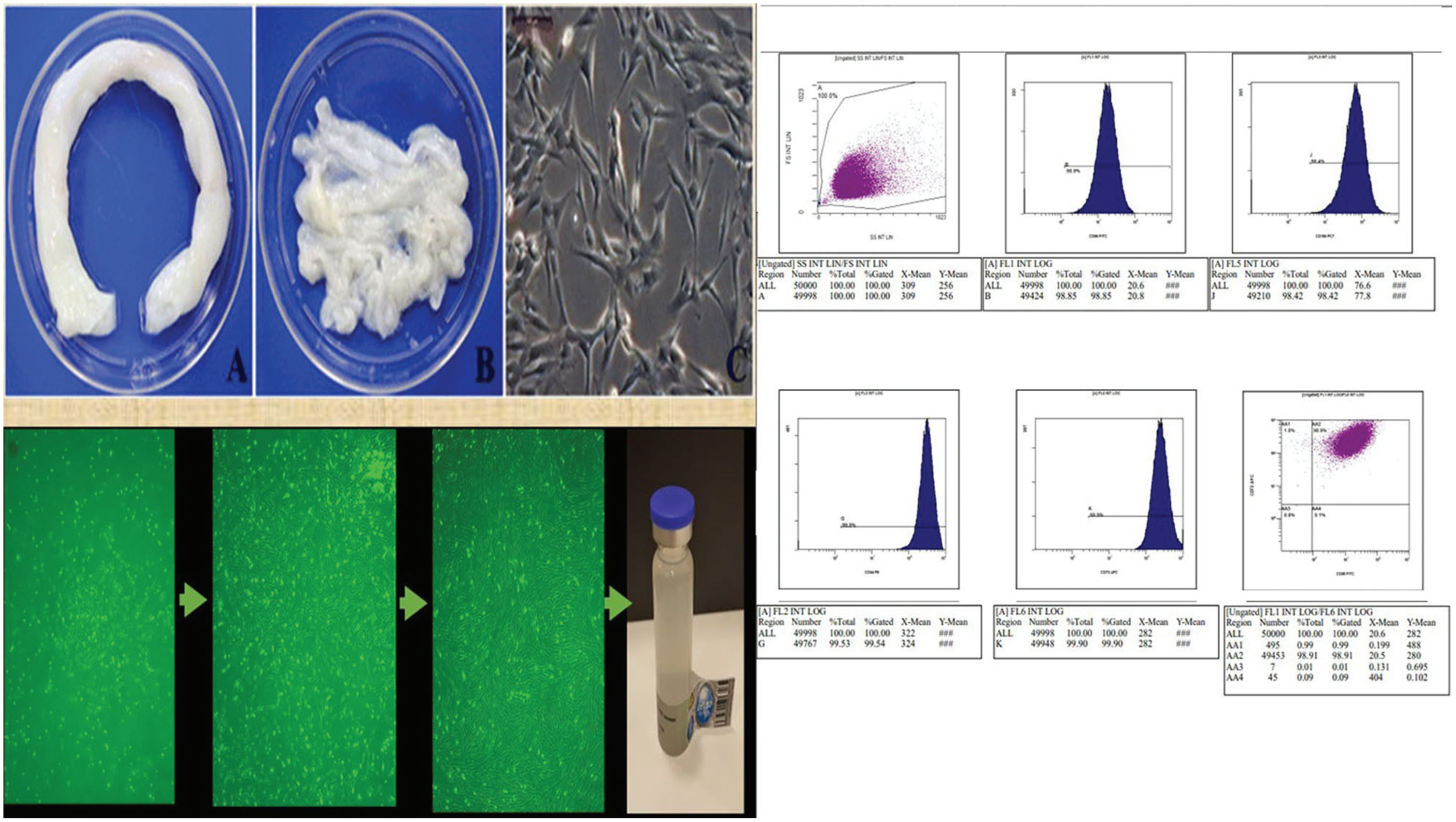
The phenotypic characterization, flow cytometric analysis, and final product of Wharton jelly derived mesenchymal stem cells. Scale bar = 200 μm.

The mesenchymal stem cells used in this study were isolated from Wharton’s jelly of the umbilical cord collected allogenicly from a single donor with the mother’s consent. The umbilical cord sample was treated following several steps. Briefly, cord tissue was washed twice with phosphate-buffered saline (Lonza), and the Wharton’s jelly part was minced using forceps and a scalpel. Minced pieces were cultivated in a cell culture dish (Greiner Bio-One) with Dulbecco’s modified Eagle’s medium F12 (DMEM)-low glucose with no L-glutamine (Biological Industries) and 10% human AB serum (Capricorn), 1% 10 000 U/mL penicillin, and 10.000 μg/mL streptomycin (Gibco). All cell preparations and cultivation procedures were conducted in a current Good Manufacturing Practice (cGMP) accredited laboratory (Onkim STEMCELL Technologies). The culture-expanded cells were cryopreserved at P3 using standard cryopreservation protocols until used in the following experiment. CryoSure-DEX40 (WAK-Chemie Medica) containing 55% dimethyl sulfoxide and 5% dextran 40 was used as a cryopreservant. The cells were characterized at the time of cryopreservation using flow cytometric analysis to determine the expression of the positive cluster of differentiation (CD) surface markers, CD90, CD105, CD73, CD44, CD29, and negative for CD34, CD45, and CD11b. Using real-time PCR (qPCR), the expressions of several genes were analyzed, such as tumor necrosis alpha (*TNF alpha*) and vimentin (*VIM*) (Fig. 1c). Quality control analyses were also completed, such as mycoplasma and endotoxin analyses (using the PCR and LAL tests combined with sterility analysis, respectively). Cells were solubilized from cryopreservation before being prepared for injection. The average cell viability for each treatment was over 90.0%, and each patient received 2–6 × 10^6^ cells in a 1.5 mL saline solution (Fig. 1).

### Injection of Umbilical Cord WJ-MSCs

**Figure 2.**
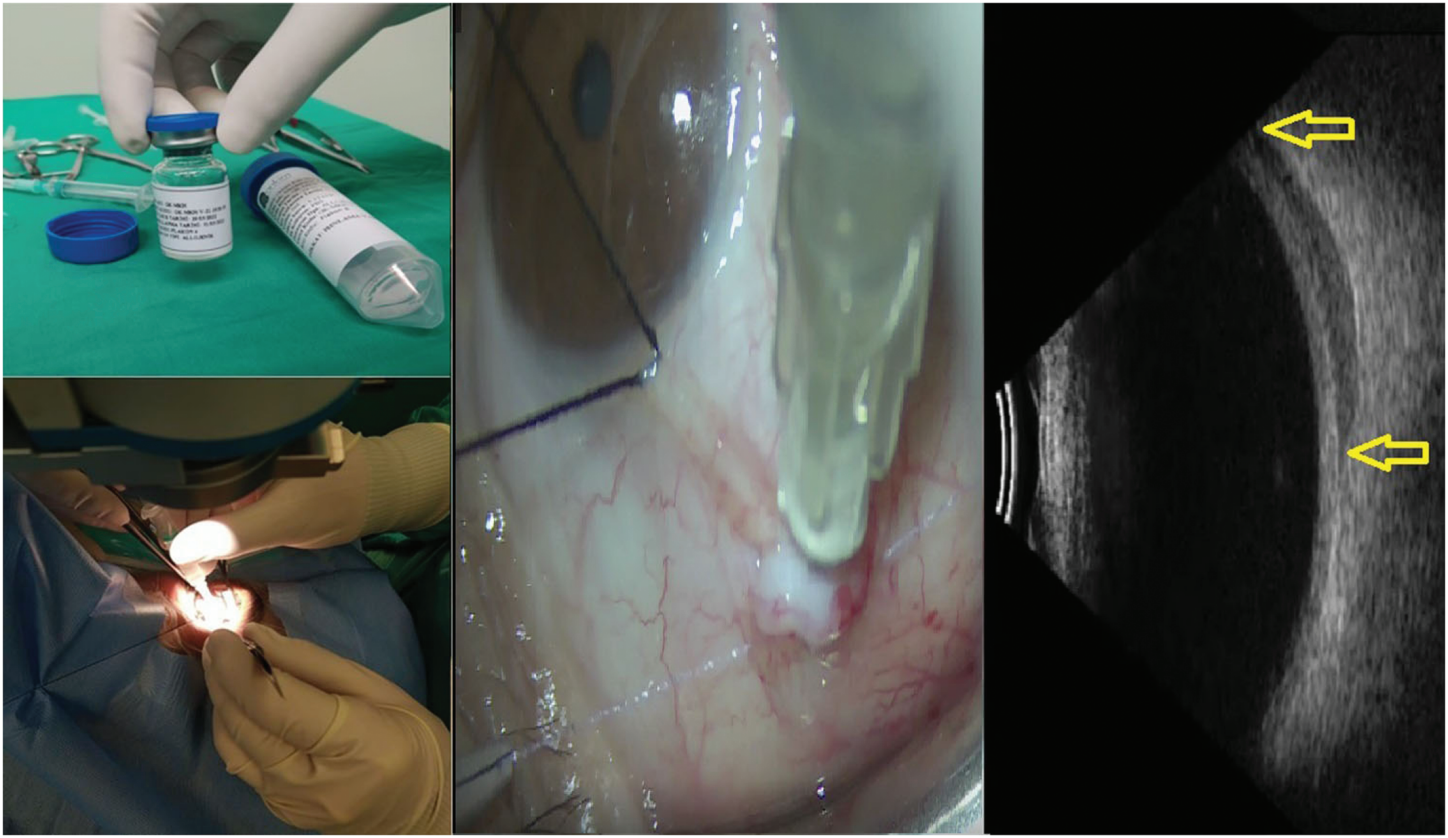
Deep subtenon injection of 5 000 000 WJ-MSC in 1.5 mL suspension and confirmation of inoculation site by orbital ultrasound.

The WJ-MSCs suspension from the culture was delivered to the operating room by cold chain and used within 24 h. A total of 1.5 mL of the WJ-MSC suspension was immediately injected into the deep subtenon space of each eye. The procedure was conducted under topical anesthesia with proparacaine hydrochloride drops (Alcaine) and sterile conditions. A 5/0 atraumatic traction suture was applied to the limbus for easy access and manipulation to the application area. A small cut was made through the conjunctiva and tenon capsule up to the sclera in the superior-temporal quadrant, 13 mm away from the limbus, to insert a 20 G subtenon curved cannula (BD, Visitec, UK). Subsequently, a 7/0 vicryl suture was passed through the conjunctiva and tenon and tied down with a loop creation. A curved subtenon cannula attached to the 2.5 cc syringe filled with 1.5 mL fluid containing stem cells was inserted through the cut and forwarded into the extraocular muscle conus until reaching the sclera; 1.5 mL of fluid was then injected. While the cannula was drawn back, a loop was tightened to prevent leakage. Postoperatively, loteprednol and tobramycin combination eye drops were given 4 times per day for 1 week, and oral amoxicillin-clavulanate (1 g) was given 2 per day for 5 days (Fig. 2).

### Retinal Repetitive Electromagnetic Stimulation (rEMS)

**Figure 3.**
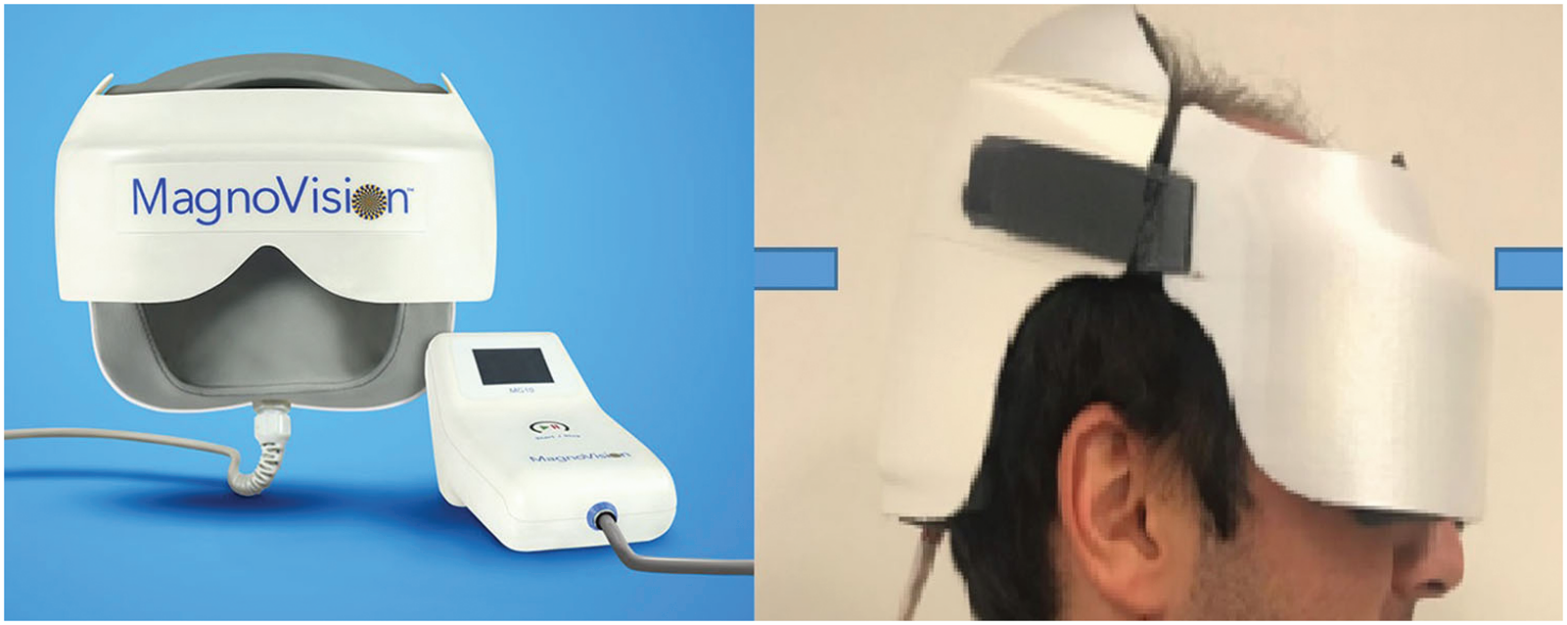
Retinal electromagnetic stimulator (Magnovision) device. Application of the helmet to stimulate the retina-optic nerve and visual pathways.

Specifically designed helmets producing high-frequency repetitive electromagnetic stimulation (Magnovision, Bioretina Biotechnology) stimulated the retinas and visual pathways in both eyes. In preclinical and clinical studies, it has been shown that the magnetic field depth for each coil is 5 cm. The location of the coils has been designed according to the anatomy of the retina, optic nerve, and visual pathways and has been demonstrated by electrophysiological tests in which only the retina/visual pathways are stimulated without any side effects. Magnovision sends sinusoidal electromagnetic waves to the nerves in vascular/neurodegenerative/ischemic retina and optic nerve diseases. The Magnovision device consists of a control unit and a helmet containing 9 coils that generate electromagnetic waves and stimulate the retina, optic nerve, and visual pathways. The electromagnetic waves generated by Magnovision cause neuronal depolarization, repolarization, and rebalancing in ion channels. The location of the coils on the helmet and the intensity, frequency, and duration of the electromagnetic field to be created have been determined as effective and safe by clinical and preclinical studies. The user or the patient cannot change effective and safe parameters. The device is designed to prevent misuse. Therapy is initiated after the helmet has been correctly positioned and adjusted on the patient’s head under the supervision of a specialist. Magnovision creates a magnetic field of 2000 miligauses with a frequency of 42 Hertz for 30 min for each therapy session. These parameters are effective and safe values determined by preclinical/clinical studies. The magnetic field intensity produced by the device is far below the safety limits recommended by the World Health Organization. The patient completes a therapy session by wearing the helmet in a sitting position for 30 min without any effort with the specially designed system for ophthalmologic use (MagnoVision; Fig. 3).

### Subjects

Retinitis pigmentosa patients were selected for the study according to the following criteria. The inclusion criteria were: RP patients of any genotype and phenotype; patients with BCVA better than 35 letters; any degree and kind of visual field loss; and patients over 18 years old.

The exclusion criteria were: the presence of glaucoma, dense cataracts and/or vitreus opacities; autoimmune retinopathy-like clinical picture; any degree of smoking; the presence of systemic neurological disease with seizure; and the presence of a cardiac pacemaker.

The RP patients were taking only OMEG3 capsules containing the same brand and the same amount of DHA and EPA as food supplements.

A total of 130 eyes of 80 RP patients who could be checked regularly, mean 36 months of the follow-up period, were included in this study. Four different groups with similar demographic characteristics were created in the cohort (Tables 1–8). The genetic mutations in the groups were not homogenous and similar (Tables 1–7).

**Table 1.** Demographic characteristics and anatomical follow-up parameters of Group 1 (management with only WJ-MSCs).

Patient no	Genetic mutation	Eye	Horizontal EZW		Vertical EZW		FAF field	
			Time0	Time1	Time0	Time1	Time0	Time1
1	RHO	R	3.56	3.58	4.62	4.64	8.1	8.2
2	RP1	L	3.21	3.27	3.36	3.38	7.9	8.0
3	EYS	R	1.86	1.55	1.15	0.88	3.1	2.5
4	PCARE	L	3.93	3.39	2.72	2.02	8.2	6.6
5	RPGR	L	1.61	0.52	1.44	0.48	0.8	0.5
6	ABCA4	R	2.74	2.34	2.49	2.00	4.9	4.0
7	C2ORF	R	4.01	3.88	4.02	3.88	15.1	14.0
8	USH2A	L	2.61	2.61	2.45	2.45	4.8	4.8
9	USH2A	L	3.91	3.90	3.92	3.90	14.9	14.8
10	RP1	L	3.90	3.47	3.86	3.43	12.2	10.4
11	PDE6B	R	3.87	3.34	3.71	3.20	9.9	8.0
12	USH2A	R	1.02	1.02	1.09	1.09	0.8	0.8
13	PDE6B	L	1.15	0.84	1.13	0.82	1.0	0.7
14	MERTK	R	2.23	1.88	2.08	1.65	3.8	2.4
15	PRPF3	L	2.18	2.18	2.14	2.14	4.1	4.1
16	RPGR	R	2.18	1.20	2.14	1.16	4.0	2.2
17	PDE6B	L	2.90	2.30	1.32	0.81	2.8	1.6
18	TULP1	L	1.42	1.40	1.25	1.24	1.1	1.0
19	USH2A	R	2.11	2.12	3.03	3.04	5.6	5.6
20	PDE6B	R	1.11	0.89	1.14	0.91	1.0	0.7
21	BBS2	R	1.15	1.15	1.06	1.06	1.1	1.1
22	PRPF3	L	2.49	2.49	2.26	2.26	4.4	4.4
23	RP1	R	2.51	2.49	2.54	2.52	5.1	5.0
24	RHO	L	3.18	3.20	3.21	3.21	8.9	8.9
25	RHO	L	3.26	3.26	3.14	3.14	9.0	9.0
26	PRPF3	L	2.11	2.11	2.29	2.29	4.0	4.0
27	BBS6	L	1.78	1.76	1.66	1.65	1.2	1.1
28	USH2A	R	1.66	1.64	1.38	1.36	1.4	1.3
29	EYS	R	1.48	1.21	1.51	1.22	1.3	0.9
30	RHO	L	4.71	4.74	4.79	4.82	15.5	15.6
31	RHO	R	4.90	4.92	3.96	3.98	15.1	16.6
	RHO	L	4.71	4.72	4.00	4.01	15.6	16.6
32	CERKL	R	2.57	2.28	2.44	2.15	4.1	3.2
	CERKL	L	1.98	1.76	1.87	1.64	2.9	2.1

Time0 (baseline), just before the Wharton Jelly derived mesenchymal stem cell injection.

Time1: 36th month after injection.

Abbreviations: EZW, ellipsoid zone width (mm); FAF, fundus autofluorescence (mm^2^); WJ-MSCs, Wharton’s jelly derived mesenchymal stem cells.

**Table 2.** Demographic characteristics and functional follow-up parameters of Group 1 (management with only WJ-MSCs).

Patient no	Eye	BCVA		FPDI		ERG magnitude	
		Time0	Time1	Time0	Time1	Time0	Time1
1	R	100	100	5	11	0.40	0.51
2	L	80	85	6	15	0.41	0.72
3	R	80	75	10	8	0.46	0.40
4	L	84	80	6	4	0.41	0.37
5	L	50	35	4	2	0.44	0.37
6	L	80	75	7	5	0.48	0.40
7	R	50	50	15	14	0.78	0.74
8	L	80	80	5	5	0.45	0.45
9	L	72	70	8	7	0.43	0.42
10	L	50	35	14	11	0.78	0.71
11	R	65	60	5	3	0.48	0.38
12	R	65	65	4	4	0.39	0.39
13	L	65	60	5	3	0.46	0.37
14	R	50	50	14	12	0.78	0.62
15	L	89	90	4	4	0.40	0.40
16	R	80	50	5	2	0.42	0.37
17	L	50	50	6	5	0.41	0.40
18	L	80	80	10	9	0.68	0.67
19	R	50	55	14	14	0.81	0.81
20	R	60	60	10	9	0.79	0.78
21	R	50	50	4	4	0.39	0.39
22	L	77	75	8	8	0.51	0.51
23	R	70	65	10	8	0.65	0.53
24	L	98	100	7	9	0.48	0.67
25	L	85	85	10	10	0.82	0.82
26	L	89	85	7	7	0.68	0.66
27	R	85	80	10	8	0.70	0.68
28	R	50	50	4	4	0.41	0.41
29	R	50	35	5	5	0.43	0.42
30	L	89	90	15	16	0.49	0.82
31	R	74	75	15	15	0.41	0.75
	L	80	80	14	14	0.41	0.72
32	R	60	50	8	5	0.45	0.37
	L	60	50	7	4	0.45	0.37

Time0 (baseline): just before the Wharton Jelly derived mesenchymal stem cell injection.

Time1: 36th month after injection.

Abbreviations: BCVA, best corrected visual acuity, (ETDRS letters); ERG magnitudes, full field flicker electroretinography magnitudes (mV); FPDI, fundus perimetry deviation index (%); WJ-MSCs, Wharton’s jelly derived mesenchymal stem cells.

**Table 3.** Demographic characteristics and anatomical follow-up parameters of Group 2 (management with only rEMS).

Patient no	Genetic mutation	Eye	Horizontal EZW		Vertical EZW		FAF field	
			Time0	Time1	Time0	Time1	Time0	Time1
1	RHO	R	6.38	6.17	6.32	6.16	35.8	34.0
		L	6.44	6.24	6.40	6.19	36.2	34.1
2	PCARE	R	2.89	2.64	2.87	2.61	8.1	6.6
		L	2.86	2.58	2.85	2.57	8.0	6.5
3	RPGR	R	1.84	1.04	1.79	1.02	2.9	1.2
		L	1.81	1.09	1.79	1.07	3.1	1.3
4	USH2A	R	4.97	4.32	4.94	4.30	25.9	25.4
		L	5.01	4.79	5.00	4.78	26.1	25.7
5	USH2A	R	4.63	4.39	4.57	4.29	22.2	20.8
		L	4.66	4.38	4.81	4.52	23.6	22.0
6	PDE6B	R	5.94	5.21	5.87	5.12	32.2	27.5
		L	6.21	5.43	6.17	5.40	32.8	27.8
7	RP1	R	3.21	2.86	3.19	2.84	10.1	9.0
		L	3.24	2.88	3.22	2.87	10.4	9.3
8	BBS1	R	2.47	2.27	2.51	2.31	6.2	5.7
		L	2.46	2.26	2.50	2.30	6.1	5.6
9	RHO	R	4.89	4.60	4.87	4.57	17.1	16.6
		L	4.82	4.53	4.81	4.52	16.8	15.8
10	USH2A	R	5.11	4.80	5.09	4.78	26.8	25.2
		L	5.16	4.85	5.12	4.80	27.1	25.5
11	BBS2	R	3.26	2.90	3.15	2.80	10.2	9.1
		L	3.24	2.89	3.21	2.86	10.3	9.2
12	RHO	R	4.51	4.34	4.50	4.34	20.1	19.2
		L	4.60	4.38	4.57	4.36	21.9	21.0
13	RHO	R	5.26	5.01	5.54	5.22	28.2	26.5
		L	5.19	4.94	5.27	4.95	27.4	25.7
14	USH2A	R	3.96	3.70	3.94	3.68	15.2	14.3
		L	3.90	3.66	3.90	3.67	15.0	14.1
15	EYS	R	2.55	2.18	2.56	2.19	6.9	5.1
		L	2.61	2.22	2.71	2.32	7.0	5.2
16	RHO	R	4.97	4.67	4.96	4.66	25.1	23.4
		L	4.86	4.57	4.89	4.59	24.0	22.4

Time0 (baseline): just before the Magnovision application.

Time1: 36th month of weekly rEMS sessions.

Abbreviations: EZW, ellipsoid zone width (mm); FAF, fundus autofluorescence (mm^2^); rEMS, repetitive electromagnetic stimulation.

**Table 4.** Demographic characteristics and functional follow-up parameters of Group 2 (management with only rEMS).

Patient no	Eye	BCVA		FPDI		ERG magnitude	
		Time0	Time1	Time0	Time1	Time0	Time1
1	R	100	90	67	60	0.87	0.80
	L	100	90	68	61	0.88	0.81
2	R	65	60	18	16	0.48	0.45
	L	65	60	17	15	0.47	0.43
3	R	50	35	5	3	0.41	0.37
	L	50	35	6	3	0.40	0.37
4	R	100	90	56	54	0.87	0.84
	L	100	90	58	56	0.89	0.87
5	R	90	85	49	47	0.78	0.76
	L	90	85	50	48	0.79	0.78
6	R	60	55	60	54	0.69	0.61
	L	60	55	62	57	0.70	0.62
7	R	75	70	20	18	0.37	0.47
	L	75	70	21	19	0.37	0.48
8	R	35	35	8	5	0.41	0.37
	L	35	35	8	5	0.44	0.40
9	R	90	95	42	40	0.87	1.03
	L	90	95	41	40	0.77	1.20
10	R	85	85	56	55	0.91	0.98
	L	85	85	58	56	0.92	1.01
11	R	50	45	20	18	0.45	0.45
	L	55	50	21	19	0.48	0.47
12	R	85	85	40	38	0.98	0.98
	L	90	85	42	40	0.99	1.20
13	R	100	100	59	59	0.87	1.14
	L	100	100	58	58	0.86	112
14	R	75	75	30	30	0.78	0.96
	L	70	70	28	28	0.74	0.90
15	R	50	35	15	10	0.47	0.37
	L	55	40	17	12	0.51	0.37
16	R	85	80	54	50	0.56	0.54
	L	80	75	51	46	0.52	0.50

Time0 (baseline): just before the Magnovision application.

Time1: 36th month of weekly rEMS sessions.

Abbreviations: BCVA, best corrected visual acuity, (ETDRS letters); ERG magnitudes, full field flicker electroretinography magnitudes (mV); FPDI, fundus perimetry deviation index (%); rEMS, repetitive electromagnetic stimulation.

**Table 5.** Demographic characteristics and anatomical follow-up parameters of Group 3 (management with WJ-MSC + rEMS combination).

Patient no	Genetic mutation	Eye	Horizontal EZW		Vertical EZW		FAF field	
			Time0	Time1	Time0	Time1	Time0	Time1
1	RHO	R	2.49	2.51	1.98	2.04	5.2	5.3
		L	2.61	2.63	2.20	2.24	5.7	5.8
2	USH2A	R	6.18	6.21	5.72	5.74	34.2	35,7
		L	6.14	6.16	5.68	5.71	34.9	36,2
3	RPGR	R	1.21	0.85	1.34	0.94	1.6	1.3
		L	1.31	0.92	1.39	0.97	1.8	1.3
4	C2ORF	R	4.72	4.68	4.61	4.57	21.6	20.5
		L	4.63	4.57	4.58	4.52	21.2	20.3
5	USH2A	R	4.02	4.02	4.06	4.05	14.9	14.8
		L	4.22	4.21	4.26	4.26	18.0	18.0
6	PDE6B	R	3.56	3.50	3.69	3.62	14.1	13.8
		L	3.63	3.55	3.74	3.68	14.6	14.2
7	RP1	R	2.05	1.97	2.03	1.96	4.7	4.5
		L	2.11	2.07	2.10	2.04	5.0	4.7
8	USH2A	R	3.14	3.14	3.18	3.18	10.0	10.0
		L	3.22	3.21	3.28	3.27	10.5	10.5
9	PDE6A	R	3.29	3.21	3.22	3.17	10.6	10.3
		L	3.17	3.10	3.28	3.21	10.4	10.1
10	USH2A	R	2.98	2.98	3.00	3.00	9.1	9.1
		L	3.11	3.11	3.08	3.08	9.6	9.6
11	BBS6	R	2.01	2.00	2.11	2.10	4.2	4.2
		L	2.10	2.08	2.06	2.05	4.4	4.3
12	RHO	R	3.56	3.58	3.54	2.56	13.0	13.1
		L	3.68	3.69	3.66	3.67	13.6	13.7
13	RHO	R	4.71	4.71	4.68	4.68	23.6	23.6
		L	4.89	4.90	4.59	4.59	24.1	24.1
14	USH2A	R	2.97	2.97	2.91	2.91	9.8	9.8
		L	2.91	2.90	2.83	2.83	9.2	9.2
15	EYS	R	1.56	1.52	1.59	1.55	2.5	2.3
		L	1.63	1.58	1.58	1.53	2.7	2.5
16	RHO	R	5.10	5.09	5.05	5.03	30.6	30.5
		L	5.21	5.19	5.11	5.09	31.9	31.7

Time0 (baseline): just before the WJ-MSC and Magnovision application.

Time1: 36th month after injection and 36th month of weekly rEMS sessions.

EZW, ellipsoid zone width (mm); FAF, fundus autofluorescence (mm^2^); rEMS, repetitive electromagnetic stimulation; WJ-MSCs, Wharton’s jelly derived mesenchymal stem cells.

**Table 6.** Demographic characteristics and functional follow-up parameters of Group 3 (management with WJ-MSC + rEMS combination).

Patient No	Eye	BCVA		FPDI		ERG magnitude	
		Time0	Time1	Time0	Time1	Time0	Time1
1	R	70	80	28	30	0.66	0.89
	L	80	85	34	36	0.70	0.91
2	R	100	110	67	71	0.63	0.96
	L	100	110	65	69	0.61	0.96
3	R	50	35	6	4	0.44	0.40
	L	50	40	8	6	0.46	0.42
4	R	85	85	54	52	0.59	0.86
	L	80	80	50	49	0.56	0.84
5	R	75	75	42	42	0.51	0.56
	L	85	85	48	49	0.55	0.61
6	R	50	45	38	37	0.45	0.51
	L	55	50	39	37	0.50	0.56
7	R	65	60	30	28	0.39	0.39
	L	65	60	31	28	0.41	0.40
8	R	75	75	36	36	0.44	0.65
	L	80	80	37	37	0.47	0.68
9	R	65	60	39	37	0.61	0.60
	L	60	55	37	35	0.60	0.58
10	R	75	75	33	33	0.63	0.74
	L	80	80	35	35	0.65	0.78
11	R	50	50	27	27	0.36	0.48
	L	50	50	28	28	0.37	0.50
12	R	80	85	36	37	0.66	0.78
	L	85	85	37	38	0.68	0.79
13	R	100	110	60	62	0.76	0.99
	L	100	110	60	62	0.75	0.98
14	R	60	60	31	31	0.37	0.74
	L	55	55	29	29	0.36	0.75
15	R	35	35	5	5	0.34	0.41
	L	40	40	8	8	0.36	0.44
16	R	110	110	61	61	0.78	0.96
	L	110	110	62	62	0.80	0.98

Time0 (baseline): just before the WJ-MSC and Magnovision application.

Time1: 36th month after injection and 36th month of weekly rEMS sessions.

BCVA, best corrected visual acuity, (ETDRS letters); ERG magnitudes, full field flicker electroretinography magnitudes (mV); FPDI, fundus perimetry deviation index (%); rEMS, repetitive electromagnetic stimulation; WJ-MSCs, Wharton’s jelly derived mesenchymal stem cells.

**Table 7.** Demographic characteristics and anatomical follow-up parameters of Group 4 (natural course).

Patient No	Genetic mutation	Eye	Horizontal EZW		Vertical EZW		FAF field	
			Time0	Time1	Time0	Time1	Time0	Time1
1	RHO	R	3.74	3.06	3.41	1.91	8.6	5.1
		L	3.46	2.71	3.61	2.74	8.9	5.4
2	PCARE	R	8.30	5.81	7.77	5.43	46.4	29.8
		L	8.30	5.81	7.97	5.57	46.8	30.1
3	RPGR	R	2.12	0.81	2.76	1.37	4.7	0.9
		L	2.71	1.32	3.01	1.56	1.9	1.3
4	USH2A	R	7.45	6.33	5.68	4.81	62.9	53.8
		L	8.24	6.96	7.98	6.81	71.8	61.5
5	PDE6B	R	3.73	2.35	2.87	2.01	9.1	6.2
		L	3.79	2.37	3.68	2.76	14.8	8.9
6	BBS2	R	1.92	1.12	1.82	1.03	3.8	1.4
		L	1.60	1.14	1.55	0.86	2.2	0.81
7	PDE6B	R	3.11	2.06	3.50	2.74	10.1	6.2
		L	3.02	2.01	3.41	2.54	9.8	4.6
8	RHO	R	5.75	4.82	5.01	4.24	26.1	16.6
		L	5.69	4.78	5.22	4.37	25.9	16.5
9	RP1	R	2.40	1.98	2.40	2.00	4.2	3.7
		L	2.70	2.14	2.60	2.06	4.8	4.0
10	USH2A	R	5.08	4.24	4.98	4.16	22.6	16.9
		L	4.98	4.19	4.71	4.14	17.2	16.6
11	BBS6	R	1.34	0.91	1.38	1.02	1.4	1.1
		L	1.29	0.87	1.30	0.90	1.2	0.84
12	TULP1	R	1.77	1.24	1.53	1.10	3.9	3.1
		L	1.54	1.12	1.70	1.25	3.2	2.9
13	RHO	R	2.86	2.41	2.77	2.38	5.6	5.0
		L	2.76	2.34	2.65	2.32	5.1	4.6
14	RHO	R	2.33	2.02	2.00	1.74	4.1	3.9
		L	1.97	1.71	1.89	1.69	3.7	3.1
15	EYS	R	1.88	1.42	1.88	1.43	3.6	2.2
		L	2.05	1.78	2.49	2.32	5.7	4.6
16	ABCA4	R	2.53	1.81	2.48	1.78	6.1	5.2
		L	2.50	1.79	2.48	1.78	6.0	5.1

Time0 (baseline): initial examination.

Time1: 36th month examination.

Abbreviations: EZW, ellipsoid zone width (mm); FAF, fundus autofluorescence (mm^2^).

**Table 8.** Demographic characteristics and functional follow-up parameters of Group 4 (natural course).

Patient no	Eye	BCVA		FPDI		ERG Magnitude	
		Time0	Time1	Time0	Time1	Time0	Time1
1	R	80	70	35	25	0.56	0.44
	L	85	80	38	29	0.60	0.50
2	R	85	70	72	51	0.72	0.64
	L	85	70	74	53	0.75	0.64
3	R	65	35	8	3	0.37	0.41
	L	50	35	5	3	0.38	0.37
4	R	100	85	80	70	0.98	0.86
	L	110	95	82	72	0.99	0.86
5	R	80	70	40	32	0.76	0.70
	L	90	75	46	37	0.80	0.72
6	R	55	40	8	5	0.41	0.38
	L	50	35	6	3	0.38	0.37
7	R	85	70	41	30	0.66	0.42
	L	80	60	39	29	0.60	0.38
8	R	110	100	58	50	0.84	0.76
	L	110	100	57	50	0.86	0.74
9	R	60	50	30	24	0.40	0.37
	L	65	50	33	26	0.42	0.39
10	R	90	80	51	40	0.56	0.50
	L	80	70	47	38	0.51	0.49
11	R	55	40	8	6	0.38	0.38
	L	50	35	6	4	0.42	0.37
12	R	45	35	11	8	0.38	0.38
	L	40	35	8	6	0.37	0.37
13	R	100	100	40	34	0.81	0.78
	L	100	100	38	32	0.80	0.78
14	R	85	85	36	29	0.78	0.72
	L	80	80	33	27	0.76	0.70
15	R	50	35	5	3	0.38	0.38
	L	60	40	8	4	0.38	0.38
16	R	90	75	33	26	0.81	0.74
	L	90	75	32	27	0.81	0.71

Time0 (baseline): initial examination.

Time1: 36th month examination.

BCVA, best corrected visual acuity, (ETDRS letters); ERG magnitudes, full field flicker electroretinography magnitudes (mV); FPDI, fundus perimetry deviation index (%).

#### Group 1

Consisted of 34 eyes of 32 RP patients treated with only WJ-MSCs, and it was applied only once following necessary preparations. After the inoculation of stem cells, the patients were followed up regularly on the 10th day, 3rd month, and every 6 months after that until 36th months. For ethical reasons, the worse eye was selected to inject the stem cells instead of both eyes.

#### Group 2

Consisted of 32 eyes of 16 RP patients treated with only rEMS. rEMS was applied with a custom-designed helmet once a week for 30 min for 36 months. Both eyes are stimulated at the same time with the specially designed system for ophthalmologic use (MagnoVision).

#### Group 3

Consisted of 32 eyes of 16 RP patients treated with the WJ-MSCs and rEMS combination. WJ-MSCs were applied first into the deep subtenon space of both eyes after necessary preparations. rEMS application was started 10 days after the WJ-MSC application with a custom-designed helmet for 30 min. WJ-MSCs were inoculated only once, and rEMS was applied regularly once a week for 30 min for 36 months. Both eyes are stimulated at the same time with the specially designed system for ophthalmologic use (MagnoVision).

#### Group 4

The natural course (control) group consisted of 32 eyes of 16 RP patients who received no treatment and were regularly followed until the 36th month. This group comprised patients who did not accept any treatment and/or were in good condition at baseline.

### Timeframe

The patients were evaluated at 2 study time points. Time 0 (T0): baseline evaluation (before the intervention) to evaluate and record the structural and functional quantitative measurements. Time 1 (T1): assessment at the 36th month to evaluate and record the structural and functional quantitative measurements.

### Primary Outcome Measure

#### Fundus Autofluorescence Surface Area (FAF-Field)

 The pattern of FAF correlates well with functional tests such as perimetry and ERG. The ring of increased autofluorescence appears to represent the border between functional and dysfunctional retinas. Metabolically active photoreceptors/RPE appear as hyperfluorescent areas in the FAF device due to the presence of lipofuscin. The FAF device calculated the FAF field automatically after marking the horizontal and vertical longest axes of the hyperfluorescent field in the posterior pole.

### Secondary Outcome Measures

#### ETDRS Visual Acuity (BCVA)

The visual acuity scores obtained from the T0 and T1 examinations were analyzed and compared using statistical tests to determine effectiveness.

#### Ellipsoid Zone Widths (EZW)

EZW showed healthy photoreceptors and was measured horizontally and vertically (HEZW and VEZW, respectively) on multimodal OCTA devices. A manual segmentation program was used for the measurement of EZW.

#### Fundus Perimetry Deviation Index (FPDI)

FPDI records were examined in the 24/2 visual field (VF) of the computerized perimetry records. The FPDI offers data explaining how many of the 100 flashing points and what percentage of the visual field could be correctly seen by the patient. For VF analysis, practice rounds were carried out 3 times before the last assessments to avoid mistakes during the test.

#### Full-Feld Multi-Luminance Flicker Electroretinography Magnitude (ERG-m)

Digital electroretinography is a non-invasive office-based objective test that measures the electrical activity of the global outer retinal cells in response to a light stimulus. ERG-m refers to the action potentials and phase deviations recorded from photoreceptors stimulated with different light intensities.

### Definition of Safety Outcome

Intraocular, intraorbital mass lesion, inflammation, fibrosis, proptosis, diplopia, afferent pupillary defect, corneal, lenticular haze, ocular allergic reactions, intravitreal, subretinal, macular hemorrhages, vitreoretinal interface alterations, retinal tears, retinal detachment (exudative, rhegmatogenous), and intraocular pressure change from baseline (≤5 mmHg) were considered to be serious adverse ocular events. Besides the routine ophthalmic examinations, OCTA multimodal imaging and B-scan orbital ultrasonography were also used to detect and confirm the presence of complications and anatomical changes during each study period examination. Systemic allergic reactions and anaphylaxis were considered to be systemic side effects.

### Statistical Methods

Descriptive statistics are presented with frequency, percentage, mean, and SD values. The Willcoxon signed rank test was used to analyze the differences in FAF-F, EZW BCVA, FPDI, and ERG-m scores according to the T0 and T1 times. The Kruskal–Wallis test was used for measurement differences between groups. The Mann–Whitney *U* test was used to compare delta changes between groups. *P*-values < 0.05 were considered statistically significant (*α* = 0.05). Analyses were done with the SPSS 25.0 package program.

## Results

Group 1 consisted of 34 eyes of 32 RP patients. Of the 32 patients, 18 were male, and 14 were female. The mean age was 39.7 (22–62 years). Group 2 consisted of 32 eyes of 16 RP patients. Of the 16 patients, 9 were male, and 7 were female. The mean age was 38.9 years (range, 22–61 years). Group 3 consisted of 32 eyes of 16 RP patients. Of the 16 patients, eight were male and eight were female. The mean age was 39.8 years (range, 22–63 years). Group 4 consisted of 32 eyes of 16 RP patients. Of the 16 patients, eight were male and eight were female. The mean age was 38.6 years (range, 23–60 years) in Group 4. The mean follow-up time between the 1st and last measurements in all groups was 36 months. There were no statistical differences between the groups regarding age and follow-up times (*P* = 0.83).

### Anatomical Assessment Parameters

#### The Mean Fundus Autofluorescence Surface Area (FAF-Field)

**Figure 4.**
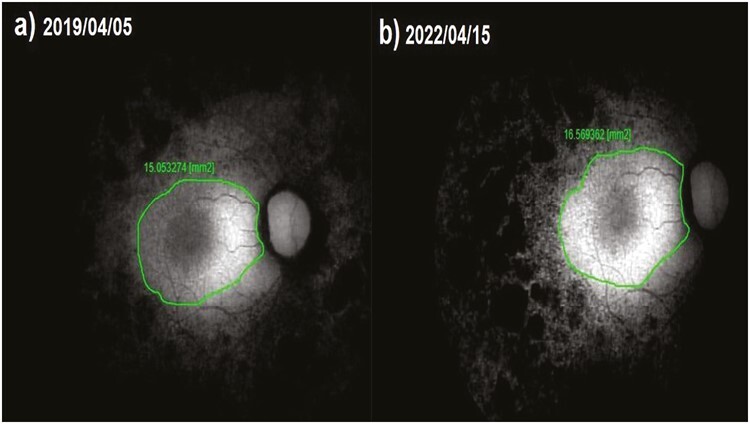
Fundus autofluorescence surface area (FAF-field) changes according to study timepoints (T0,T1) in the eye treated with only WJ-MSC. Note the change in FAF-field values (Table 1, patient 31: right eye). (**a**) Before application, FAF-field 15.1 mm^2^. (**b**) At 36th month, FAF-field 16.6 mm^2^.

**Figure 5.**
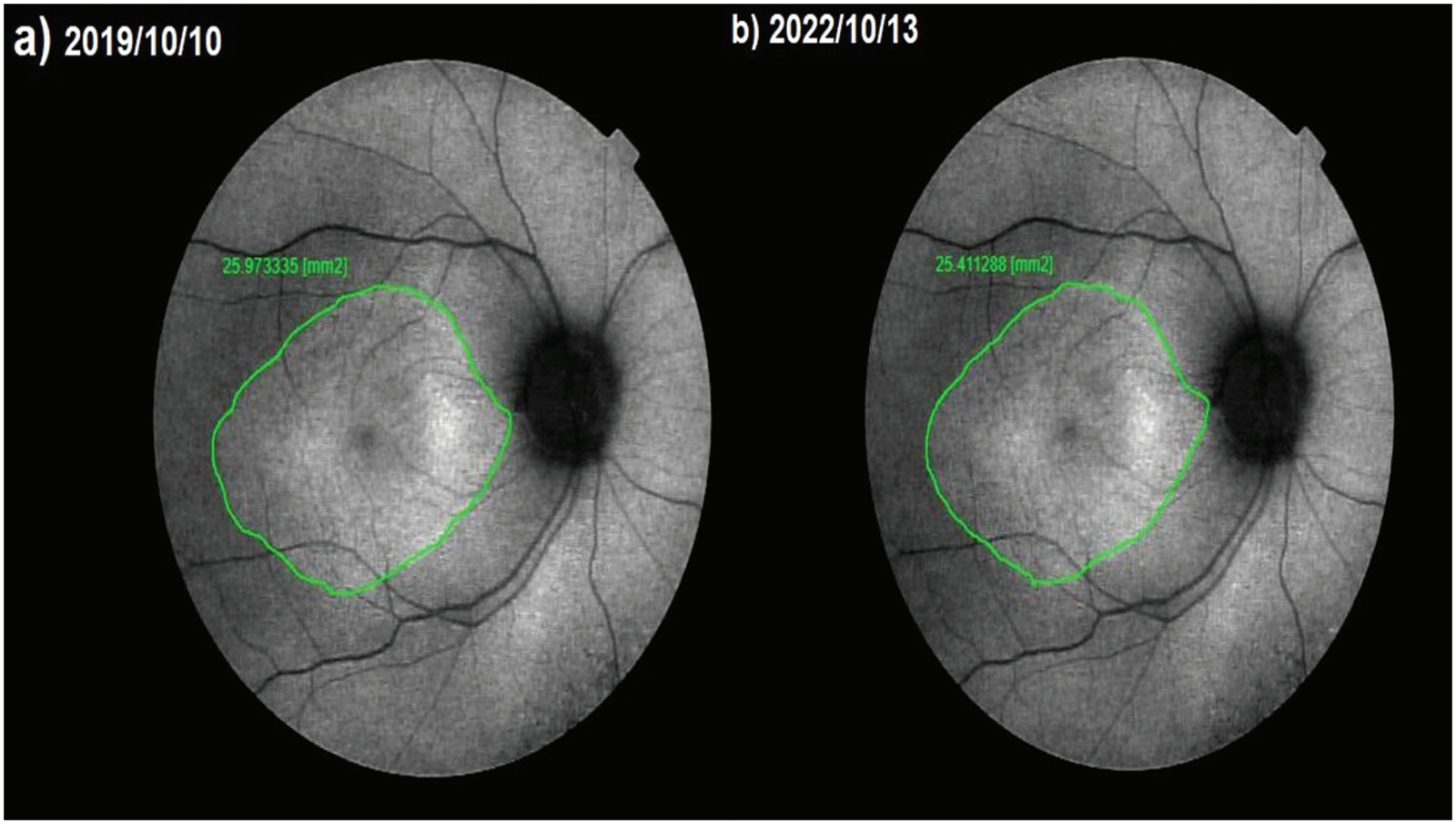
Fundus autofluorescence surface area (FAF-field) changes according to study timepoints (T0, T1) in the eye treated with only rEMS Note the change in FAF-field values (Table 3, patient 4: right eye). (**a**) Before application, FAF-field 25.9 mm^2^. (**b**) At 36th month, FAF-field 25,4 mm^2^.

**Figure 6.**
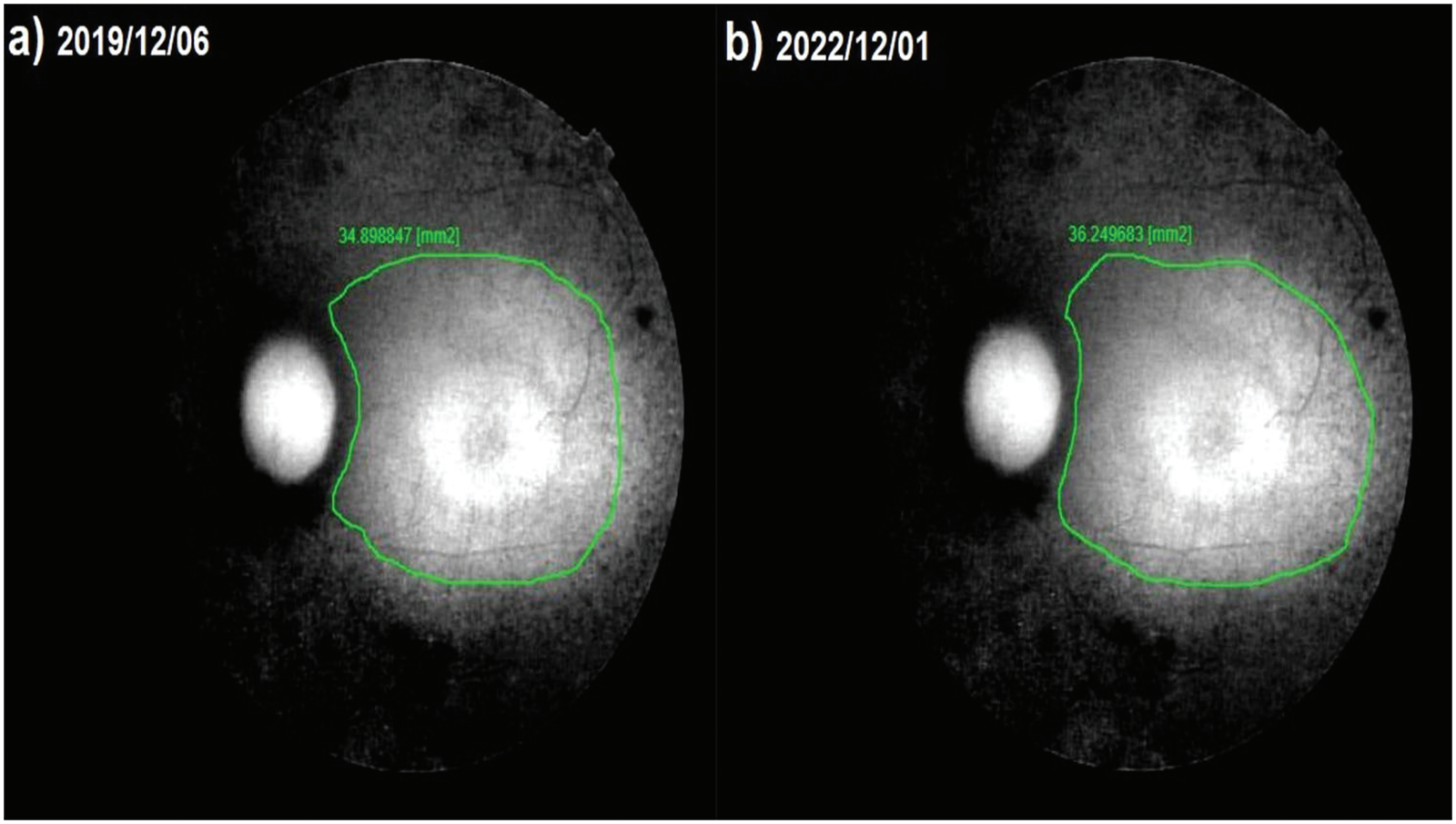
Fundus autofluorescence surface area (FAF-field) changes according to study timepoints (T0,T1) in the eye treated with combination of WJ-MSC + rEMS. Note the change in FAF-field values (Table 5, patient 2: left eye). (**a**) Before application, FAF-field 34.9 mm^2^. (**b**) At 36th month, FAF-field 36.2 mm^2^.

**Figure 7.**
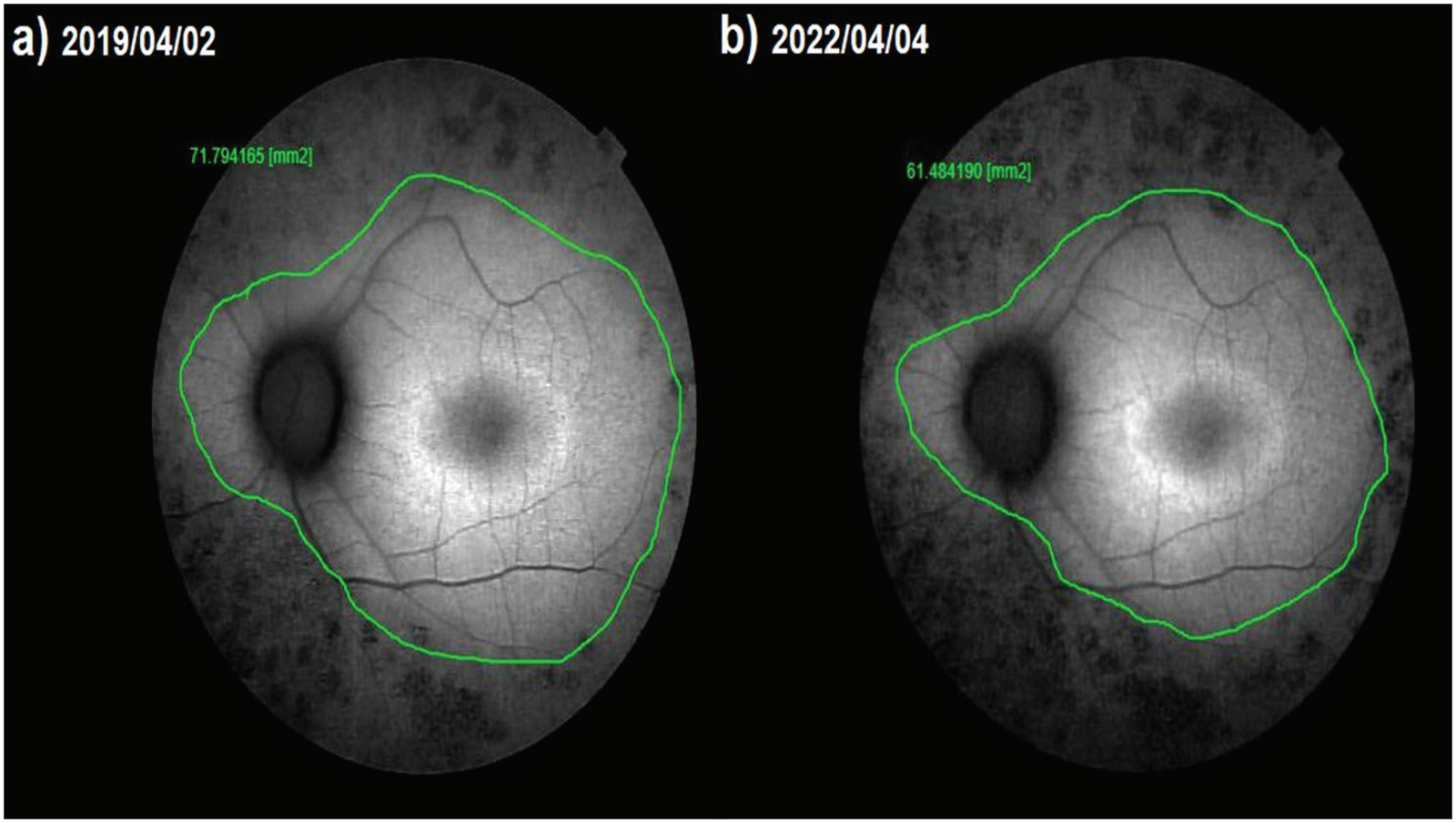
Fundus autofluorescence surface area (FAF-field) changes according to study timepoints (T0,T1) in the untreated eye (natural course). Note the change in FAF-field values (Table 7, patient 4: left eye). (**a**) Initial examination, FAF-field 71.8 mm^2^. (**b**) At 36th month, FAF-field 61.5 mm^2^.

This value was 5.99 mm^2^ in Group 1 before the only WJ-MSC applications and 5.60 mm^2^ after the procedures at the 36th month (*P* =0.01). In Group 2, the FAF field was 18.4 mm^2^ at the first measurement and 16.9 mm^2^ after only rEMS applications at the 36th month (*P* = 0.04). In Group 3, the FAF field was 13.35 mm^2^ before combining WJ-MSC and rEMS applications and 13.28 mm^2^ at the last examination in the 36th month (*P* = 0.41). In Group 4, the FAF field was 14.13 mm^2^ at the initial examination and 10.37 mm^2^ at the last examination in the 36th month (*P* = 0.01). The FAF-field delta change of the groups can be ranked as Group 3 (0.07) < Group 1 (0.39) < Group 2 (1.50) < Group 4 (3.76) (Tables 1–7; Figs. 4a-7b).

#### The Mean Horizontal Ellipsoid Zone Width (m-HEZW)

**Figure 8.**
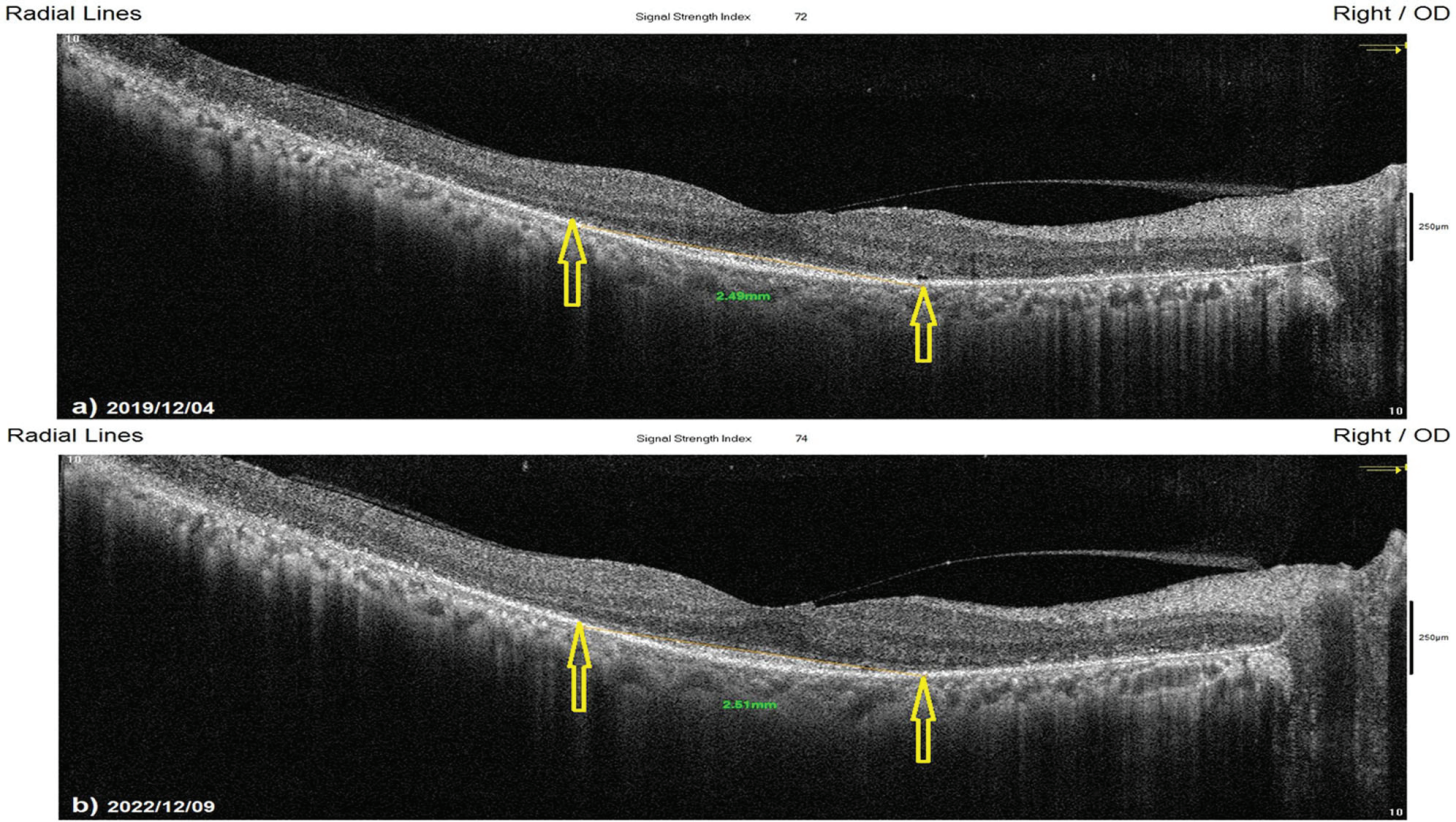
Horizontal EZW changes according to study timepoints (T0,T1) in the eye treated with combination of WJ-MSC + rEMS. Note the change in Horizontal EZW values (Table 5, patient 1: right eye). (**a**) Before application, EZW 2.49 mm. (**b**) At 36th month, EZW 2.51 mm.

This value was 2.65 mm in Group 1 before the only WJ-MSC applications and 2.45 mm after the procedures at the 36th month (*P* = 0.01). In Group 2, the m-HEZW was 4.18 mm at the first measurement and 3.84 mm after only rEMS applications at the 36th month (*P* = 0.01). In Group 3, the m-HEZW was 3.35 mm before combining WJ-MSC and rEMS applications and 3.28 mm at the last examination in the 36th month (*P* = 0.06). In Group 4, the m-HEZW was 3.53 mm at the initial examination and 2.67 mm at the last examination in the 36th month (*P* = 0.01). The m-HEZW delta change of the groups can be ranked as Group 3 (0.04) < Group 1 (0.20), Group 2 (0.34) < Group 4 (0.86) (Tables 1–7; Fig. 8a and 8b).

#### The Mean Vertical Ellipsoid Zone Width (m-VEZW)

**Figure 9.**
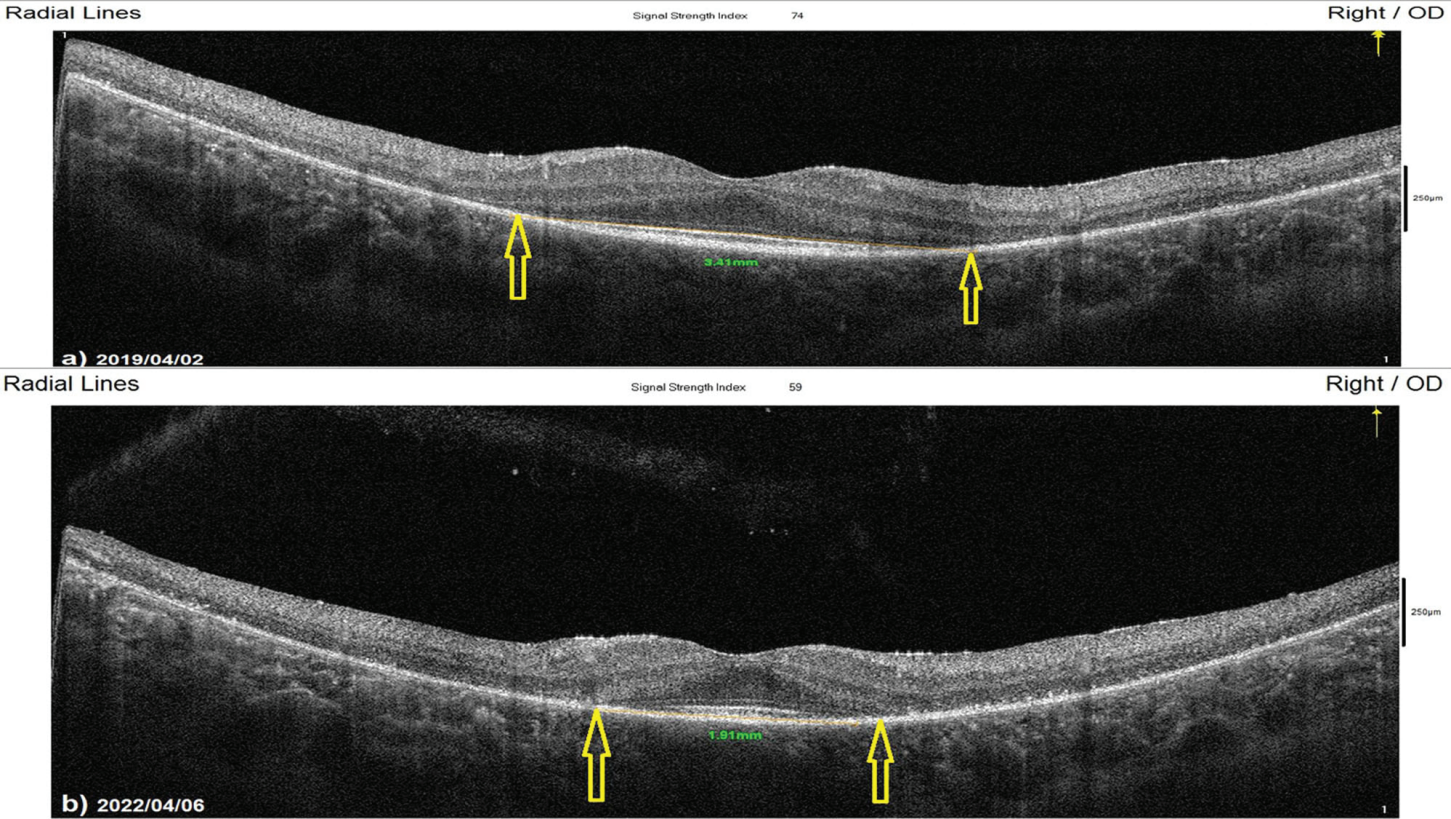
Vertical EZW changes according to study timepoints (T0,T1) in untreated eye (natural course). Note the change in vertical EZW values (Table 7, patient 1: right eye). (**a**) Initial, EZW 3.41 mm. (**b**) At 36th month, EZW 1.91 mm.

This value was 2.51 mm in Group 1 before the only WJ-MSC applications and 2.31 mm after the procedures at the 36th month (*P* = .01). In Group 2, the m-VEZW was 4.18 mm at the first measurement and 3.83 mm after only rEMS applications at the 36th month (*P* = .01). In Group 3, the m-VEZW was 3.32 mm before combining WJ-MSC and rEMS applications and 3.25 mm at the last examination in the 36th month (*P* = .06). In Group 4, the m-VEZW was 3.39 mm at the initial examination and 2.59 mm at the last examination in the 36th month (*P* = .01). The m-VEZW delta change of the groups can be ranked as Group 3 (0.07) < Group 1 (0.20) < Group 2 (0.35) < Group 4 (0.80) (Tables 1–7, Fig. 9a and 9b).

### Functional Assessment Parameters

#### The Mean Best Corrected Visual Acuity (m-BCVA)

This value was 70.5 ETDRS letters in Group 1 before the only WJ-MSC applications and 66.9 letters after the procedures at the 36th month (*P* = 0.04). In Group 2, the m-BCVA was 74.8 letters at the first measurement and 70.0 letters after only rEMS applications at the 36th month (*P* = 0.04). In Group 3, the m-BCVA was 72.5 letters before combining WJ-MSC and rEMS applications and 72.6 letters at the last examination in the 36th month (*P* = 0.87). In Group 4, the m-BCVA was 76.8 letters at the initial examination and 64.8 letters at the last examination in the 36th month (*P* = 0.01). The m-BCVA delta change of the groups can be ranked as Group 3 (-0.1) < Group 1 (3.6) < Group 2 (4.8) < Group 4 (12.0) (Tables 2–8).

#### The Mean Fundus Perimetry Deviation Index (m-FPDI)

**Figure 10.**
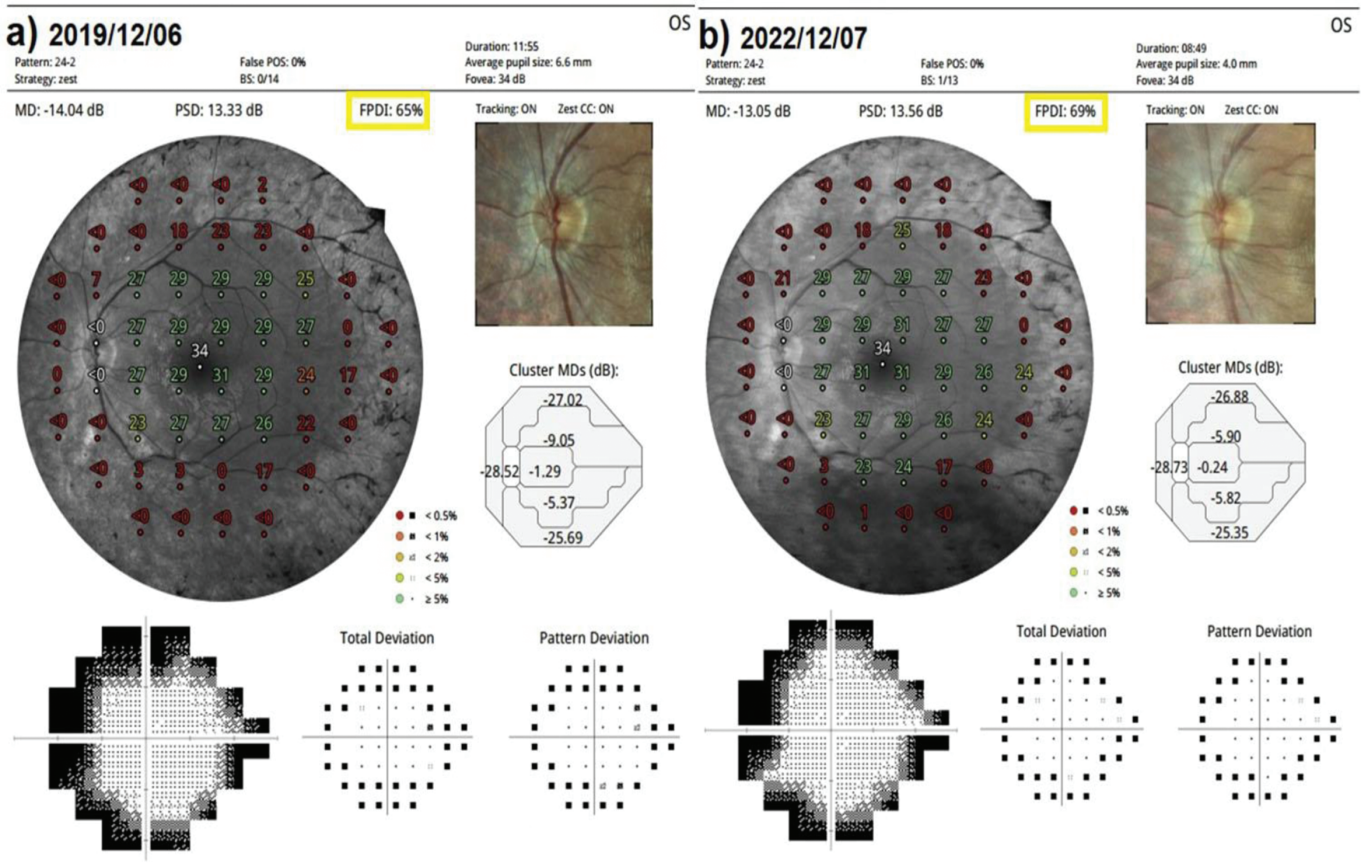
Visual field FPDI changes according to study timepoints (T0,T1) in the eye treated with combination of WJ-MSC + rEMS. Note the change in FPDI values (Table 6, patient 2: left eye). (**a**) Before application, FPDI 65%. (**b**) At 36th month, FPDI 69%.

**Figure 11.**
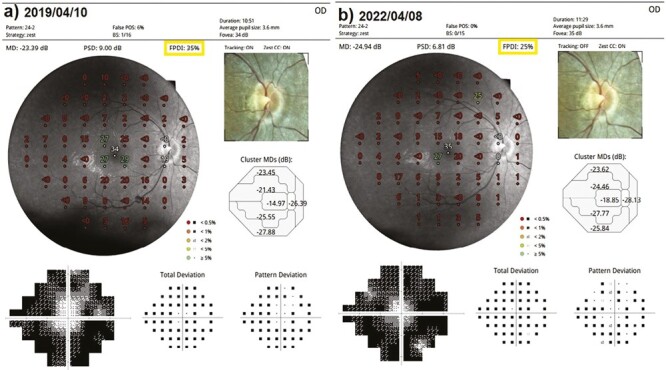
Visual field FPDI changes according to study timepoints (T0,T1) in untreated eye (natural course). Note the change in FPDI values (Table 8, patient 1: right eye). (**a**) Initial, FPDI 35%. (**b**) At 36th month, FPDI 25%

This value was 8.3% in Group 1 before the only WJ-MSC applications and 7.8% after the procedures at the 36th month (*P* = 0.03). In Group 2, the m-FPDIwas 37.6% at the first measurement and 35.0% after only rEMS applications at the 36th month (*P* = 0.04). In Group 3, the m-FPDI was 37.5% before the combination of WJ-MSC and rEMS applications and 37.5% at the last examination in the 36th month (*P* = 0.96). In Group 4, the m-FPDI was 34.6% at the initial examination and 27.4% at the last examination in the 36th month (*P* = 0.01). The m-FPDI delta change of the groups can be ranked as Group 3 (0.01) < Group 1 (0.50) < Group 2 (2.66) < Group 4 (7.25) (Tables 2–8; Figs. 10a-11b).

#### The Mean Full-Field Multiluminance Electroretinography Magnitude (ERG-m)

**Figure 12.**
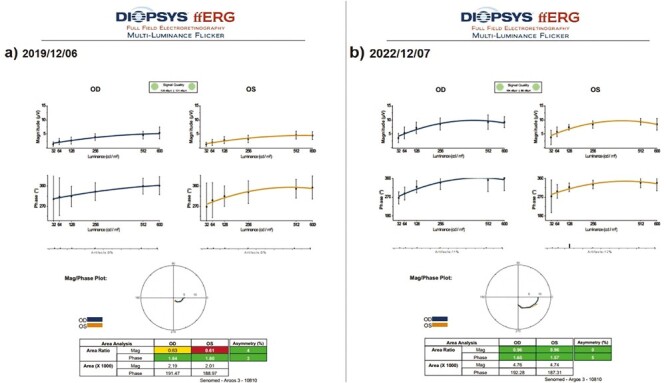
Full field ERG magnitude changes according to study timepoints (T0,T1) in the eye treated with combination of WJ-MSC + rEMS. Note the change in ERG-m values (Table 6, patient 2: right and left eye). (**a**) Before application, ERG-m right 0.63 mV, left 0.61 mV. (**b**) At 36th month, ERG-m right 0.96 mV, left 0.96 mV.

**Figure 13.**
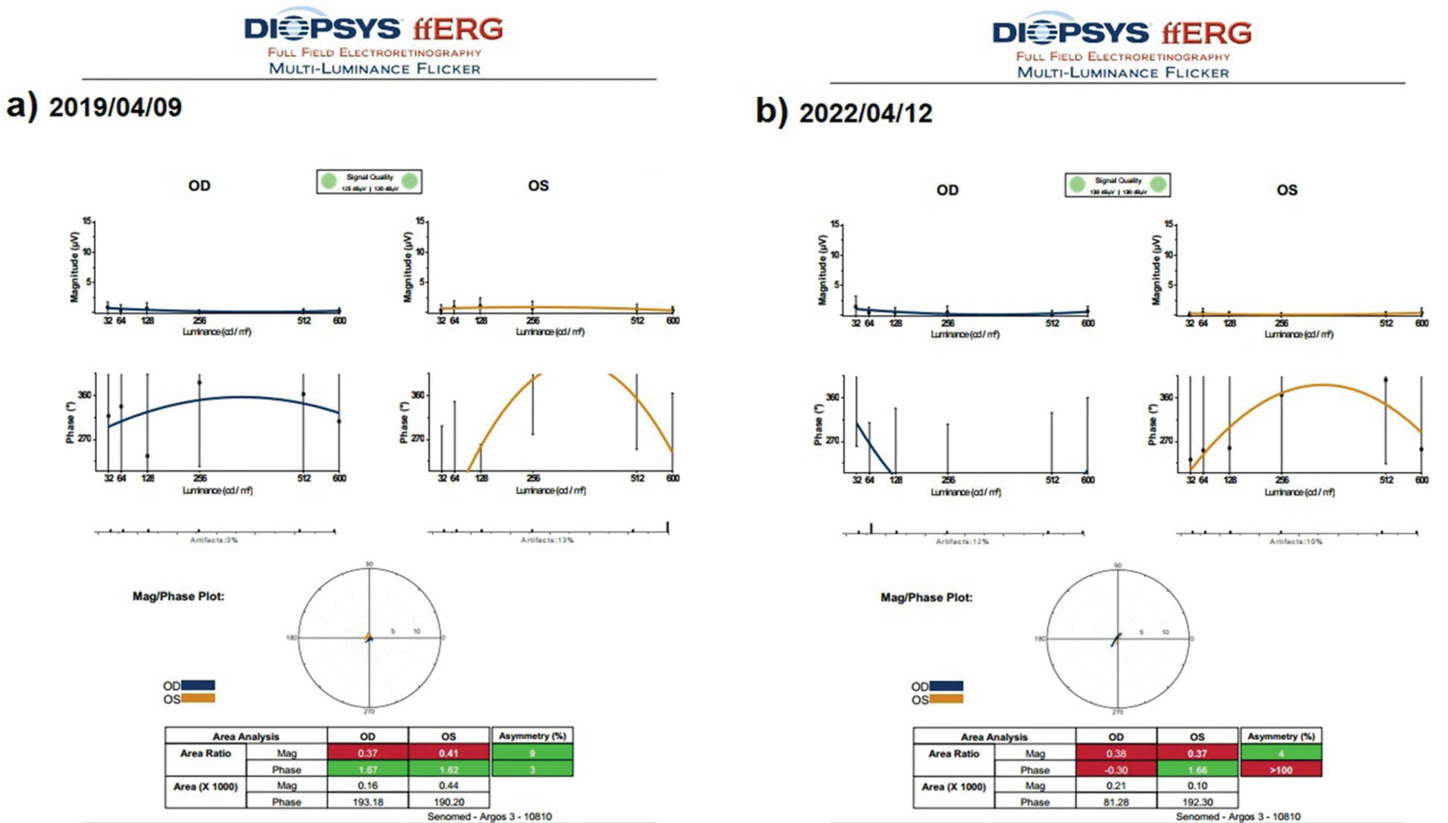
Full field ERG magnitude changes according to study timepoints (T0,T1) in untreated eye (natural course). Note the change in ERG-m values (Table 8, patient 3: right and left eye). (**a**) Initial, ERG-m right 0.37 mV, left 0.41 mV. (**b**) At 36th month, ERG-m right 0.38 mV, left 0.37 mV.

This value was 0.53 mV in Group 1 before the only WJ-MSC applications and 0.54 mV after the procedures at the 36th month (*P* = 0.45). In Group 2, the ERG-m was 0.67 mV at the first measurement and 0.71 mV after only rEMS applications at the 36th month (*P* = 0.12). In Group 3, the ERG-m was 0.55 mV before combining WJ-MSC and rEMS applications and 0.69 mV at the last examination in the 36th month (*P* = 0.01). In Group 4, the ERG-m was 0.61 mV at the initial examination and 0.50 mV at the last examination in the 36th month (*P* = 0.01). The ERG-m delta change of the groups can be ranked as Group 3 (−0.14) > Group 2 (−0.04), Group 1 (−0.01), and Group 4 (0.06) (Tables 2–8, Figs. 12a and 13b).

When Groups 1, 2, 3, and 4 were compared using the Kruskal–Wallis test according to the delta changes, the combined application of WJ-MSC and rEMS significantly increased all assessment parameters (Table 9**).**

**Table 9.** Comparison of assessment parameters before the applications and at the end of the 3-year follow-up period between 4 groups.

To-T1	Wj-Msc^1^	Magnovision^2^	Wj-Msc + Magnovision^3^	Control^4^	P***	Difference**
	X ± s.s.	X ± s.s.	X ± s.s.	X ± s.s.		
ΔHorizontal EZW	0.20 ± 0.05	0.34 ± 0.08	0.04 ± 0.01	0.86 ± 0.21	0.01*	4 > 1.2 > 3
ΔVertical EZW	0.20 ± 0.04	0.35 ± 0.09	0.07 ± 0.02	0.80 ± 0.22	0.03*	4 > 1.2 > 3
ΔFAF field	0.39 ± 0.11	1.50 ± 0.41	0.07 ± 0.02	3.76 ± 1.02	0.01*	4 > 2 > 1 > 3
ΔBCVA	3.59 ± 0.97	4.84 ± 1.31	−0.16 ± -0.04	12.04 ± 3.25	0.01*	4 > 1.2 > 3
ΔFPDI	0.50 ± 0.14	2.66 ± 0.72	0.01 ± 0.00	7.25 ± 1.96	0.01*	4 > 2 > 1 > 3
ΔERG magnitude	−0.01 ± 0.00	−0.04 ± −0.01	−0.14 ± -0.04	0.06 ± 0.02	0.02*	3 > 1,2,4

***Kruskall-Wallis test; **Mann-Whitney *U* test; *0.05 statistically significant.

BCVA, best corrected visual acuity, (ETDRS letters); ERG magnitudes, full field flicker electroretinography magnitudes (mV); EZW, ellipsoid zone width (mm); FAF, fundus autofluorescence (mm^2^); FPDI, Fundus perimetry deviation index (%).

In Group 1, all patients stated that they felt transient foggy vision in the first 40 days and flashes of light between 40 days and 60 days. Patients in Groups 2 and 3 who underwent rEMS did not report these complaints. No serious ocular or systemic adverse events were encountered in any group related to WJ-MSC and rEMS applications at the 36th month.

## Discussion

The light coming from the objects to the eye is refracted from the transparent media of the eye and focused on the retina. The retina consists of 2 parts, the sensory retina and the retina pigment epithelium. Photoreceptor cells (rod and cones) constitute the outer layer of the neurosensory retina, where photochemical reactions begin. Photosensitive proteins such as opsin/rhodopsin, encoded in the sensory retina, initiate certain chemical reactions called the visual cycle. These reactions open and close ion channels in neurons, creating action potentials with neuronal depolarization and repolarization; these electrical signals in the outer retina are transmitted to the optic nerve, visual pathway, and visual cortex. There are an average of 120 million photoreceptor cells in the retina. About 100 million rod cells are responsible for night vision and peripheral vision. About 20 million cone cells are responsible for sharp vision and color vision.^17^ Retinitis pigmentosa is a genetic disease group characterized by progressive loss of photoreceptor cells and degeneration of the outer retinal layers. The inheritance pattern can be autosomal dominant, autosomal recessive, X-linked, mitochondrial, or spontaneous mutations.^1-3^ Genetic mutations in RP can affect retina-specific and non-specific structural and functional protein levels. Retina-specific proteins are opsin, rhodopsin, RPE65, and RPGR cilia proteins.^18^ In classical RP, genetic mutations primarily impair the functions of rod cells. Structural and functional protein deficiency causes rod cells to enter the dormant phase and then apoptosis.^19^ Therefore, the rate of disease progression is different in each inheritance pattern. Complete deprivation of retinal-specific proteins presents clinically with severe vision loss at an early age or rapid progression in advanced age. Non-specific protein-coding genetic mutations in RP impair the transfer of glucose into cells, the formation of energy molecules such as ATP-GTP, and the phagocytosis and digestion of metabolic wastes, such as lipofuscin.^18^ Some genetic mutations in RP can lead to the accumulation of miscoded and misfolded proteins in the RPE and photoreceptor cells, which can lead to complications such as intense inflammation, macular edema, epiretinal membrane, and cataracts. In this group, apoptosis triggered by inflammation causes rapid disease progression.^20^ Editing each mutant gene with gene therapy is not cost-effective, and the diversity of inheritance patterns makes gene therapy extremely difficult. For this reason, gene-agnostic treatments, such as stem cell therapies and optogenetic therapy, are being studied intensively.^1,21^

Wharton’s jelly-derived mesenchymal stem cells have a high paracrine effect, and the exosomes contain neurotrophins, growth factors, micro-RNA, and mitochondrial components. These growth factors are neural growth factor (NGF), insulin-like growth factor (IGF), brain-derived neurotrophic factor (BDNF), and ciliary neurotrophic factor (CNTF). Growth factors and neurotrophins activate by binding to tyrosine kinase receptors on the RPE and photoreceptor membranes. Peptides secreted by WJ-MSCs accelerate glucose uptake, oxidative phosphorylation, mitochondrial functions in neurons, phagocytosis, and digestion of metabolic wastes; provide neural homeostasis; and regulate neural metabolism and oxidative energy cycle. Mitochondrial fragments in exosome content also increase ATP-GTP levels. Exosomes also contain anti-inflammatory cytokines and peptides that regulate B and T lymphocyte functions, such as prostaglandin E and transforming growth factor. This anti-inflammatory effect of WJ-MSCs contributes to preventing apoptosis triggered by inflammation.^22-26^ Due to all these mechanisms above, many preclinical and clinical studies have shown that WJ-MSCs can reactivate dormant phase photoreceptors, improve retinal functions, prevent photoreceptor apoptosis, and slow disease progression in RP and other retinal degenerations.^25-29^ In the long-term follow-up of RP patients, whose results we have previously published, we have had objective observations that exosome production slows down or stops within 1-2 years in some cases.^25,26^ Telomere length indicates that WJ-MSCs can survive for 3-7 years in the appropriate medium and microenvironment.^30,31^ Theoretically, WJ-MSCs have a lifespan of at least 3 years. To investigate whether exosome production can be stimulated, we combined the application of WJ-MSC with repetitive electromagnetic stimulation.

Clinical studies show that electromagnetic stimulation therapy is effective and safe in some neurological and psychiatric diseases such as medication-resistant depression, stroke, Parkinson’s disease, Alzheimer’s disease, or Multiple Sclerosis.^9^ Electromagnetic stimulation accelerates or slows down neural transmission through ion channels, depending on the frequency and magnetic field intensity in neurons. Excitation and inhibition in neurons are defined as the neuromodulation effect of rEMS.^10,32^ Electromagnetic stimulation accelerates mitochondrial and neuronal metabolism by increasing the affinity of tyrosine kinase receptors to growth factors in neuronal cell membranes.^33^ This effect also increases the passage of drug molecules into the neurons and is defined as iontophoresis.^34,35^ The iontophoresis feature of rEMS is used in the treatment of major depression.^36^ Clinical studies show that electromagnetic stimulation can have effective results in ophthalmology and neurological and psychiatric diseases. Neuromodulation, increase in growth factor activity, and iontophoresis properties are used in neurodegenerative and ischemic retina/optic nerve diseases in ophthalmology.^11-16^ Electromagnetic therapy has increased regenerative growth factor levels at the retina and optic nerve. It also increases synaptic conduction, blood flow, and growth factor-receptor sensitivity.^37,38^ The device can be used in chronic eye diseases of the retina and optic nerve for a period and frequency determined by the physician without causing any side effects. Electromagnetic therapy can be used as a complementary therapy to increase the effect of stem cells, growth factors, and some other therapeutic drugs, or in some cases, it can be used alone. Mesenchymal stem cells inoculated into deep subtenon space secrete growth factors into the degenerated microenvironment. Magnovision, specifically developed for ophthalmologic use, can synergistically enhance the degranulation of growth factor-containing vesicles in mesenchymal stem cells through ion channels.^37^

The rate of disease progression in RP is related to the inheritance pattern. In the retina, 2 units of each protein type are produced by 2 alleles. In autosomal dominant (AD) inheritance, 1 of the 2 alleles is mutant. One unit of native protein can be produced by the normal allele. Therefore, the rate of progression is relatively slow. The average annual photoreceptor loss in AD RP is reported to be approximately 5% in the literature.^39-43^ Both alleles are mutant in autosomal recessive (AR) inheritance, so the disease progresses faster. The average annual photoreceptor loss in AR RP is reported to be approximately 10% in the literature.^39,44^ Retina-specific functional proteins are encoded on the X chromosome. Therefore, X-linked RP progresses much faster in male cases. The average annual photoreceptor loss in X-linked RP is reported to be approximately 15% in the literature.^42,45^ The ability of the FAF device is used to map naturally and pathologically occurring fluorophores in the posterior pole. Brighter areas represent regions of increased lipofuscin density. The ring of increased autofluorescence appears to represent the border between functional and dysfunctional retinas. The FAF pattern, a non-invasive, easy-to-use functional test, correlates well with functional tests such as perimetry and ERG.^46-48^ In our study, Group 4 was followed as a natural course. The sample included all inheritance patterns and the most common classical RP mutations in Türkiye.^49^ In the context of FAF measurements, the 3-year photoreceptor loss rate was found to be 27% on average. This progression rate was consistent with the literature.^39-48^ Group 1, which underwent only WJ-MSC, consisted of classical RP cases with similar inheritance and genetic mutations. The 3-year photoreceptor loss rate was 7%. The reduction in photoreceptor loss rate confirms the paracrine effects of subtenon-injected WJ-MSCs. Group 2, which underwent only Magnovision, consisted of classical RP cases with similar inheritance and genetic mutations. The 3-year photoreceptor loss rate was 9%. The reduction in the photoreceptor loss rate confirms that rEMS activates ion channels and delays the entry of photoreceptors into the dormant phase. Preventing the entry of photoreceptors into the dormant phase may also reduce the rate of apoptosis in the long run. Group 3, which underwent a combination of WJ-MSC and Magnovision, consisted of classical RP cases with similar inheritance and genetic mutations. The 3-year photoreceptor loss rate was 0.5%. WJ-MSC and rEMS synergistically can significantly reduce photoreceptor loss.

Several hypotheses explain the synergistic effects of the combination of WJ-MSC and rEMS. rEMS can stimulate the degranulation of mesenchymal stem cell exosomes.^33^ Stimulation of degranulation may cause an increased concentration of neurotrophins, growth factors, anti-inflammatory cytokines, and mitochondrial components in the outer retinal microenvironment.^50^ Another hypothesis is that the paracrine effects of WJ-MSC may decrease 1 year after administration.^4-8^ rEMS can periodically stimulate WJ-MSCs for exosome production and degranulation. The periodic slow-release stimulating effect of rEMS may also explain the synergistic effect.^51-54^ Another hypothesis is the iontophoresis effect of rEMS. The subtenon space is an immunoprotective partially avascular region and is a suitable medium for WJ-MSCs.^55,56^ There are studies in the literature in which WJ-MSCs are applied more invasively and traumatically as suprachoroidal, subretinal, and intravitreal. Complications such as inflammation, retinal tear, fibrosis, and photoreceptor loss due to surgical trauma have been reported in these invasive applications.^56-58^ WJ-MSC is effective when administered intravenously, but it was not preferred due to the risk of systemic complications such as thrombosis, embolism and requiring more mesenchymal stem cells.^56^ To avoid these complications, we prefer deep subtenon injection that does not enter the globe and can be relatively non-invasive. Our goal is to deliver the exosome content to the outer retinal layers. Delivery of WJ-MSCs to the outer retinal layers is not intended, which is inappropriate in terms of RP pathophysiology. Exosome contents secreted by WJ-MSCs in the subtenon space pass into the choroidal matrix via scleral pores. Molecules with a molecular weight of less than 50 kD can pass through the scleral pores by passive diffusion. By varying their surface electrical charge, larger molecules can pass through scleral pores by iontophoresis. rEMS can alter the electrical charges of scleral pores and peptides, allowing more exosome contents to enter the choroidal matrix. This situation is frequently used in neuropsychiatry as electromagnetic iontophoresis increases drug entry into the cell in treatment-resistant cases. Growth factors and neurotrophins that pass into the choroidal matrix reach the RPE and photoreceptors via specific tyrosine kinase receptors. Electromagnetic iontophoresis can increase receptor affinities and efficiency.^34-36^ Electromagnetic iontophoresis may also explain the synergistic effect of WJ-MSC and Magnovision. The magnetic field intensity used is far below international standards and is within safe limits.^59^ No adverse effects were observed due to Magnovision.

In the context of visual field deterioration, there was a loss of FPDI values during the follow-up by 21% in natural course group. But in the combined treatment group, visual field values remained almost the same. In this study, magnitude values of multi-luminance ERG increased during the follow-up period with all kinds of treatment measures; in the control group magnitude values decreased significantly at the end of the 36th month.

In the natural course group without any treatment, all functional and anatomical measurements deteriorated significantly with time.

Combined use of WJ-MSCs and rEMS is the most effective treatment modality to slow and maintain the retinitis pigmentosa’s progression significantly during the 36 months, compared to the application of only WJ-MSCs or only rEMS. Only stem cells were more effective than the only rEMS regarding the slowing rate. Most of the treated patients express improvement in daily life during the follow-up assessments, but it has to be measured and confirmed with the “Quality of Life Index.”

In Group 1, in which WJ-MSCs treatment was used alone, all subjects expressed foggy vision or light flashes within the first 3 months for a certain period of time. These complaints were not expressed by any of the patients in the group receiving the combination of WJ-MSC and Magnovision. The stabilization of cell membranes explains foggy vision in these RP patients. Slow depolarization and repolarization of ion channels lead to slower neurotransmission, delayed conduction, and hazy vision.^60^ Flashes of light in RP are defined as neuronal noise caused by unnecessary excitation of ion channels. Direct stimulation of the visual system other than light causes phosphenes or photopsias produced by incorrect depolarization and repolarization of ion channels.^61^ In applying WJ-MSC alone, the rapid action of the released anti-inflammatory cytokines and the cortisone-like membrane stabilization may explain the foggy vision. Reactivation of photoreceptors in the dormant phase but inappropriate synaptic neurotransmission with bipolar cells may explain light flashes. Magnovision’s excitability of ion channels with physiological frequency may prevent fogging and flashing complaints that develop after WJ-MSC application.^53^

The research has some limitations. In some cases, the anatomical data and functional data were incompatible. FAF-field, EZW, and visual field are not correlated in some cases. In these cases, the comparison of homogeneous genetic mutation types, detection of functional protein mutations, and inflammatory mechanisms such as the complement system should be addressed in separate research. For ethical reasons, the worse eye is generally selected for stem cell injection.

## Conclusion

Retinitis pigmentosa is a genetic and neurodegenerative disease characterized by progressive loss of photoreceptors and outer retinal layers, eventually leading to total blindness. The combination of Wharton’s jelly-derived mesenchymal stem cells and Magnovision can significantly slow the progression of the disease in comparison to natural progression rate for 3 years in appropriate cases.

## Data Availability

The datasets generated during and/or analyzed during the study are available from the corresponding author on reasonable request. All authors had full access to all of the data in this study and take complete responsibility for the integrity of the data and accuracy of the data analysis.
